# Transcriptome Analysis of Pre-Storage 1-MCP and High CO_2_-Treated ‘Madoka’ Peach Fruit Explains the Reduction in Chilling Injury and Improvement of Storage Period by Delaying Ripening

**DOI:** 10.3390/ijms22094437

**Published:** 2021-04-23

**Authors:** Han Ryul Choi, Min Jae Jeong, Min Woo Baek, Jong Hang Choi, Hee Cheol Lee, Cheon Soon Jeong, Shimeles Tilahun

**Affiliations:** 1 Department of Horticulture, Kangwon National University, Chuncheon 24341, Korea; hanryul192@kangwon.ac.kr (H.R.C.); jmj717@naver.com (M.J.J.); minwoo100@kangwon.ac.kr (M.W.B.); choijh107@kangwon.ac.kr (J.H.C.); yaya9459@kangwon.ac.kr (H.C.L.); 2 Interdisciplinary Program in Smart Agriculture, Kangwon National University, Chuncheon 24341, Korea; 3 Agriculture and Life Science Research Institute, Kangwon National University, Chuncheon 24341, Korea; 4 Department of Horticulture and Plant Sciences, Jimma University, Jimma 378, Ethiopia

**Keywords:** peach fruit, transcriptome, CO_2_, storage, 1-MCP, softening

## Abstract

Cold storage of peach fruit at low temperatures may induce chilling injury (CI). Pre-storage 1-MCP and high CO_2_ treatments were reported among the methods to ameliorate CI and reduce softening of peach fruit. However, molecular data indicating the changes associated with pre-storage 1-MCP and high CO_2_ treatments during cold storage of peach fruit are insufficient. In this study, a comparative analysis of the difference in gene expression and physico-chemical properties of fruit at commercial harvest vs. stored fruit for 12 days at 0 °C (cold-stored (CS), pre-storage 1-MCP+CS, and pre-storage high CO_2_+CS) were used to evaluate the variation among treatments. Several genes were differentially expressed in 1-MCP+CS- and CO_2_+CS-treated fruits as compared to CS. Moreover, the physico-chemical and sensory data indicated that 1-MCP+CS and CO_2_+CS suppressed CI and delayed ripening than the CS, which could lead to a longer storage period. We also identified the list of genes that were expressed commonly and exclusively in the fruit treated by 1-MCP+CS and CO_2_+CS and compared them to the fruit quality parameters. An attempt was also made to identify and categorize genes related to softening, physiological changes, and other ripening-related changes. Furthermore, the transcript levels of 12 selected representative genes from the differentially expressed genes (DEGs) in the transcriptome analysis were confirmed via quantitative real-time PCR (qRT-PCR). These results add information on the molecular mechanisms of the pre-storage treatments during cold storage of peach fruit. Understanding the genetic response of susceptible cultivars such as ‘Madoka’ to CI-reducing pre-storage treatments would help breeders release CI-resistant cultivars and could help postharvest technologists to develop more CI-reducing technologies.

## 1. Introduction

The family Rosaceae covers a wide range of fruit types (drupes, pomes, achenes, hips, follicles, and capsules) with various growing habits ranging from herbaceous to cane, bush and tree forms [1]. The drupe fruits’ species (peaches, apricots, almonds, plums, and cherries) are important crops worldwide. For instance, 24.45 million tons of peaches and nectarines were produced per year in 2018, out of which the Republic of Korea took a share of 205,741 Mt [2]. Peaches (*Prunus persica* L.) provide a range of vitamins, minerals, fiber, carotenoids, phenolics, and anthocyanin for a healthy diet [1,3,4,5]. In particular, the level of phenolics and antioxidant activity of red-fleshed peaches is comparable to that measured in blueberries, and its antioxidant activity is best correlated with phenolic content among peach cultivars [3,6]. 

Peach is a climacteric fruit, and the initiation and continuation of its ripening process are accelerated by ethylene [7,8,9]. While ripening is desirable to fulfill the customers’ needs, a prolonged storage period is equally important for the marketing and distribution of fleshy fruits [10]. Peach fruit is vulnerable to damage after harvest due to its soft tissues [11] and deteriorates quickly at ambient temperature [12]. One of the most common methods to slow the ripening processes and to extend the postharvest life of peach fruit is low-temperature (0–5 °C) storage [13,14]. However, prolonged storage of peach fruit at low temperatures can cause chilling injury (CI), a physiological disorder leading to a reduction in value. CI in peach causes wooly fruit that lacks juice and is undesirable to consumers [12,15]. Internal and external browning, reddish discoloration and breakdown of the flesh, decay, and the loss of normal ripening are some symptoms of CI [10,16]. Hence, methods to ameliorate CI can essentially contribute to the fruit industry. Jin et al. [17] reported that treating peach fruit with 0.5 μL L^−1^ 1-methylcyclopropene (1-MCP) can prevent CI. Similarly, a significant reduction in CI and softening of peach fruit was reported following pre-storage high-CO_2_ treatment [10]. Although the above studies have reported the effect of 1-MCP and high CO_2_ treatment on the CI and physicochemical characteristics of peach fruit, molecular data indicating the changes during storage of peach fruit is insufficient. Hence, in this study, we treated ‘Madoka’ peach fruit with 1-MCP and high CO_2_, and stored the fruit at 0 °C for 12 days. The treated fruit were compared with the control to study the candidate genes related to CI and ripening of peach fruit. 

## 2. Results

### 2.1. Assembly and Annotation

**Figure 1:**
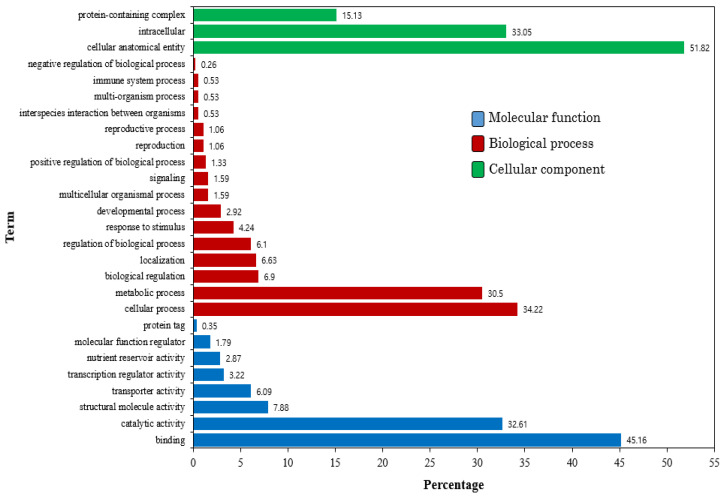
The percentage of genes in different GO sub-categories.

Mapping and sequencing results are summarized in Table 1 and Table 2. Novaseq 6000 platform and Illumina Truseq Stranded mRNA library prep kit were used for sequencing. Eight ‘Madoka’ peach fruit transcriptome datasets were generated in this study. The datasets were fruit at commercial harvest (control), CS, 1-MCP+CS, and CO_2_+CS, all in duplicate. A total of 44.05, 62.46, 60.05, and 62.41 million average reads were generated from the control, CS, CS+1-MCP, and CS+CO_2_, respectively. From the total reads, 41.52, 59.57, 58.01, and 59.84 million were mapped with high mapping rate (>94%) to the reference *Prunus persica* (GCA_000346465.2) from the control, CS, CS+1-MCP, and CS+CO_2_, respectively. We identified a total of 26,411 unigenes in the transcriptome contigs, and the identified unigenes were classified into three functional categories. From the identified unigenes, we assigned 258 genes to molecular function, 530 to biological processes, and 129 to cellular component. Further subcategories were also observed within each main category of the GO classification scheme, and the dominant subcategories from each main category are presented in Figure 1. Higher percentage of genes in molecular function were mainly involved in binding and catalytic activity. Moreover, cellular processes and metabolic processes encompasses 64% of the genes in biological processes. Similarly, the genes in cellular component were mainly categorized as cellular anatomical entity, intercellular component, and protein-containing complex (Figure 1).

### 2.2. DEGs in the Comparison of CS, 1-MCP+CS, and CO_2_+CS vs. Control Peach Fruit

**Figure 2:**
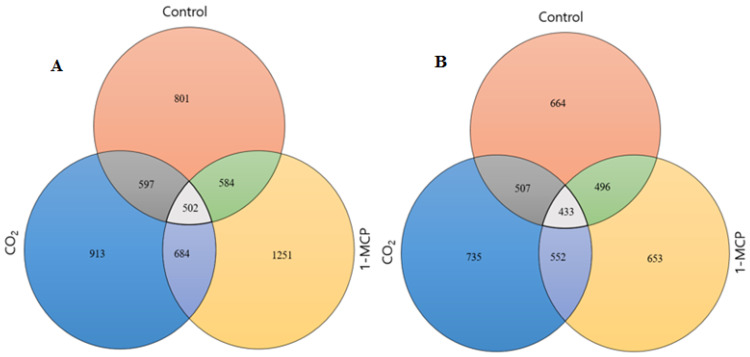
Numbers of commonly and exclusively expressed (**A**) upregulated and (**B**) downregulated genes based on twofold change and *p*-value < 0.05 during the comparison of control (control vs. CS), 1-MCP (control vs. 1-MCP+CS), and CO_2_ (control vs. CO_2_+CS) ‘Madoka’ peach fruit after 12 days storage at 0 °C.

**Figure 3:**
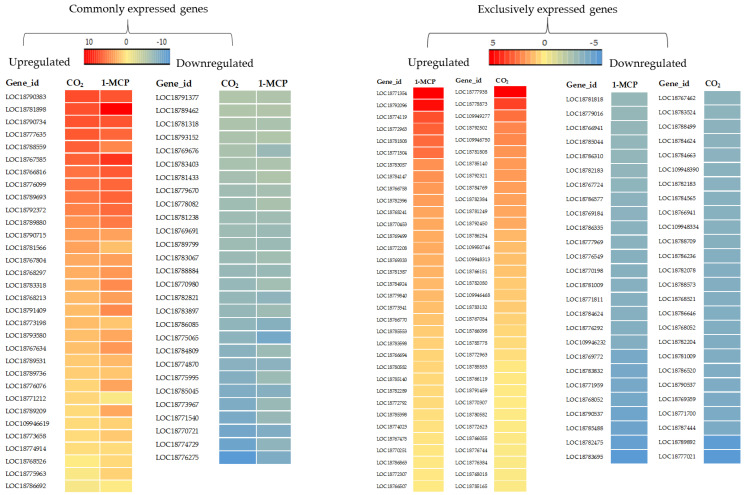
Heat map of commonly and exclusively expressed and identified DEGs in the comparison of CS vs. CO_2_+CS and 1-MCP+CS ‘Madoka’ peach fruit after 12 days storage.

Differentially expressed genes (DEGs) were compared on the basis of twofold change and *p* value < 0.05 during the comparison of control to CS, 1-MCP+CS, and CO_2_+CS. Venn diagrams summarize the number of overlapping differentially expressed genes in the comparison of CS, 1-MCP+CS, and CO_2_+CS vs. control (Figure 2). A total of 1465, 1904, and 1648 unigenes were differentially expressed during the comparison of CS, 1-MCP+CS, and CO_2_+CS, vs. control ‘Madoka’ peach fruit, respectively. From the differentially expressed unigenes, 801 (55%), 1251 (66%), and 913 (55%) were upregulated, and 664 (45%), 653 (34%), and 735 (45%) were downregulated in the CS, 1-MCP+CS, and CO_2_+CS fruit, respectively. More unigenes were expressed due to the combined effects of CS and pre-storage treatment of ‘Madoka’ peach fruit with 1-MCP and CO_2_ as compared to the CS (Figure 2). Commonly and exclusively expressed and identified DEGs in the comparison of CS vs. CO_2_+CS and 1-MCP+CS ‘Madoka’ peach fruit were summarized by heat map (Figure 3). A long list of identified, commonly and exclusively expressed DEGs that might attract researchers for more detailed work are reported in this study (Tables S1–S3). Attempts were also made to briefly discuss and relate some of the identified genes into different ripening-related parameters.

### 2.3. CI, Firmness, Total Pectin, PG Activity, and Related Genes

**Figure 4:**
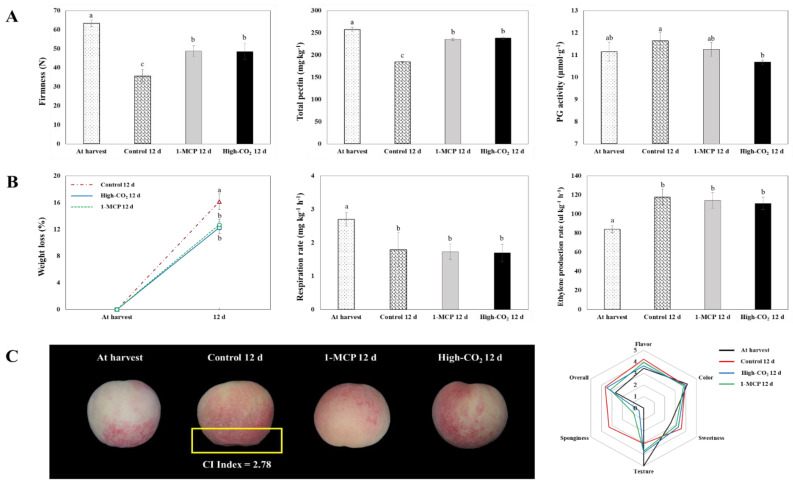
Firmness, total pectin, PG activity (**A**); weight loss, respiration rate, ethylene production rate (**B**); and fruit appearance, CI index, and sensory qualities (**C**) of ‘Madoka’ peach fruit at harvest and after 12 days storage at 0 °C with pre-storage 1-MCP and high CO_2_ treatments or without treatment (control). Data for firmness, CI index, and sensory quality are presented as a mean of 10 biological replicates ± standard errors. Total pectin, PG activity, weight loss, respiration rate, and ethylene production rate are presented as a mean of three replicates ± standard errors. Different letters on the bars indicate significant difference between treatments with Duncan’s multiple range test at *p* ≤ 0.05.

Pre-storage treatment of ‘Madoka’ peach fruit with 1-MCP and high CO_2_ was effective in delaying CI and the ripening process, as observed on the firmness, total pectin, and PG activity (Figure 4A). Quality parameters and the transcriptome analysis in this study were compared on the 12-d storage on the basis of the observation of CI in the control fruit (Figure 4C). CI was not observed in the fruit treated with pre-storage 1-MCP and high CO_2_ combined with cold storage while the untreated cold-stored fruit showed a CI index of 2.78% and higher sponginess after 12-d storage (Figure 4C). 

The firmness, one of the most important quality indices for fruits, in CS fruit decreased from 63.31 N at harvest to 35.60 N on day 12, in contrast to 48.72 and 48.47 N in 1-MCP and high-CO_2_-treated fruit on the same day, respectively. Total pectin content also showed the same trend with firmness. However, PG activity was higher in the CS fruit as compared to pretreated fruit (Figure 4A). 

Figure 3 shows the commonly and exclusively expressed and identified DEGs during the comparison of CS vs. 1-MCP+CS and CO_2_+CS. Tables S1–S3 also show gene expression levels and the details of their gene ID, mRNA_accession, protein accession, and gene descriptions. In this study, the combined effect of pre-storage high CO_2_ and 1-MCP treatment with CS reduced the rate of solubilization of pectin and PG activity (Figure 4A) as compared to CS. Moreover, *beta-amylase 3, chloroplastic (BAM3)* was commonly upregulated in both 1-MCP+CS and CO_2_+CS as compared to CS. Moreover, *vegetative cell wall protein gp1* and *the probable aquaporin PIP2-5* were upregulated due to the 1-MCP+CS (Figure 3, Tables S1 and S2). Nevertheless, the genes that encode cell wall hydrolytic enzymes such as *endoglucanase 6*, *pectin acetylesterase 12*, *probable galacturonosyltransferase 15*, and *endo-1,3; 1,4-beta-D-glucanase* were also commonly downregulated in both 1-MCP+CS and CO_2_+CS. Similarly, the genes encoding *expansin-A6* and *expansin-A4* were also downregulated in both 1-MCP+CS and CO_2_+CS (Figure 3, Table S1). The *probable pectate lyase 8* was also downregulated due to CO_2_+CS (Figure 3, Table S3). 

### 2.4. Weight Loss, Respiration and Ethylene Production Rates, and Related Genes

Weight loss of CS fruit was significantly higher than 1-MCP+CS and CO_2_+CS on the 12-d storage (Figure 3B). However, respiration and ethylene production rates were not significantly different between CS, 1-MCP+CS, and CO_2_+CS. The results imply that storage at low temperature could be efficient in reducing respiration and ethylene production rates, but the weight loss was significantly hindered by pre-storage 1-MCP and CO_2_ treatment. This could have been the possible reason for firmness and pectin content being maintained (Figure 3A). Moreover, we observed the significant down regulation of *1-aminocyclopropane-1-carboxylate oxidase homolog 1*, *transcript variant X1* in both 1-MCP+CS and CO_2_+CS as compared to CS (Figure 3, Table S1).

### 2.5. Stress-Related Genes Due to 1-MCP+CS, and CO_2_+CS Treatments

Following cold storage of high-CO_2_- and 1-MCP-treated fruits, genes related to stress response exhibited significant upregulation as compared to CS. Stress-related transcription factors such as *ethylene-responsive transcription factor ERF113*; *ethylene-responsive transcription factor ERF071*; and *AP2-like ethylene-responsive transcription factor TOE3*, *transcript variant X2* were upregulated in response to 1-MCP+CS and CO_2_+CS as compared to CS (Figure 3, Table S1). *Thaumatin-like protein 1*, *transcription factor MYB1R1*, *zinc finger protein ZAT10*, and *bZIP transcription factor 60* were also upregulated in response to 1-MCP+CS and CO_2_+CS as compared to CS. Moreover, abiotic stress acclimation genes such as *cold shock protein CS66* and *NAC transcription factor 29* were upregulated due to 1-MCP+CS treatment (Figure 3, Table S2). However, *12-oxophytodienoate reductase 3 (OPR3)* and *glyceraldehyde-3-phosphate dehydrogenase (GAPDH)* were commonly downregulated due to 1-MCP+CS and CO_2_+CS treatments as compared to CS (Figure 3, Table S1).

### 2.6. Color and Other Changes and the Related Genes

*Anthocyanidin 3-O-glucosyltransferase 2 (3GT)*, *glutathione S-transferase F12*, *3-hydroxy-3-methylglutaryl-coenzyme A reductase 1(HMGR)*, and *methanol O-anthraniloyltransferase (AMAT)* were commonly downregulated due to 1-MCP+CS and CO_2_+CS treatments (Figure 3, Table S1). Similarly, the genes that encode *dihydroflavonol 4-reductase (DFR)* and *chalcone–flavonone isomerase (CHI)* were downregulated commonly in 1-MCP+CS and CO_2_+CS, and CO_2_+CS, respectively (Figure 3, Tables S1 and S3).

### 2.7. Verification of DEGs by qRT-PCR

**Figure 5:**
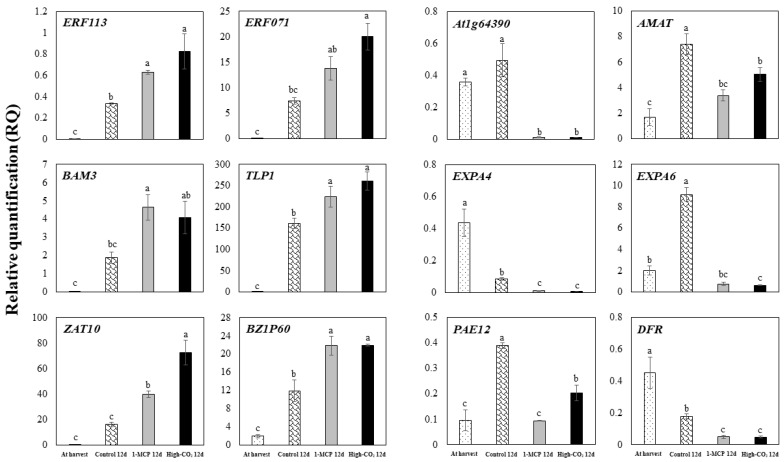
Transcript accumulation of the selected DEGs of ‘Madoka’ peach fruit by qRT-PCR for the comparison of fruit at harvest and after 12 days storage at 0 °C with pre-storage 1-MCP and high-CO_2_ treatments or without treatment (control). Vertical bars represent standard errors of the means (n = 3). Different letters on the bars indicate significant difference between treatments with Duncan’s multiple range test at *p* ≤ 0.05.

The transcript levels of 12 selected representative genes (*ERF113*, *ERF071*, *BAM3*, *TLP1*, *ZAT10*, *BZIP60*, *At1g64390*, *AMAT*, *EXPA6*, *EXPA4*, *PAE12*, and *DFR*) were confirmed by qRT-PCR (Table 3 and Figure 5). Figure 5 shows transcript accumulation of the selected DEGs of ‘Madoka’ peach fruit by qRT-PCR for the comparison of control to CS, 1-MCP+CS, and CO_2_+CS. The expression trends of RNA-seq and qRT-PCR were quite similar for the observed 12 genes (Figure 5, Table S1). These results indicated that the expression patterns of these representative genes tested in the RNA-seq assay coincided with the results of qRT-PCR analysis, suggesting that the RNA-seq data are reliable. *ERF113*, *ERF071*, *BAM3*, *TLP1*, *ZAT10*, and *BZIP60* showed a significant higher expression in both 1-MCP+CS and CO_2_+CS as compared to CS. However, *At1g64390*, *AMAT*, *EXPA6*, *EXPA4*, *PAE12*, and *DFR* showed significantly lower expression in both 1-MCP+CS and CO_2_+CS as compared to CS. 

## 3. Discussion

Peach (*Prunus persica* L.) fruit are highly perishable and deteriorate quickly at ambient temperature. Cold storage is a common strategy to slow the ripening process and extend shelf life [13,14]. However, low-temperature storage could cause improper ripening and CI to the susceptible cultivars such as ‘Madoka’. Pre-storage treatments such as 1-MCP and high CO_2_ are known to reduce CI [10,17]. Hence, we treated ‘Madoka’ peach fruit with 1-MCP and high CO_2_ and stored them at 0 °C. The treated fruit were compared with the control to study the physico-chemical changes during cold storage and the associated genetic responses. 

In this study, CI was not observed in 1-MCP+CS and CO_2_+CS, while the CS fruit showed a CI index of 2.78% after 12-d storage. According to Lee [10], moderate and severely injured fruit with CI index > 20% are not commercially acceptable due to the injury of the mesocarp surface on the opposite sides of the stone. Maruyama et al. [18] reported that the expression of genes encoding starch degrading enzymes such as *beta-amylase 3, chloroplastic (BAM3)* increased specifically under cold conditions. In this study, *BAM3* was commonly upregulated in both 1-MCP+CS and CO_2_+CS as compared to CS, indicating that pre-storage 1-MCP and high CO_2_ could induce more CI resistance. 

The ripening of peach fruit involves degradation or modification of cell wall [19]. During fruit ripening, there could be solubilization and depolymersation of pectin, which in turn contributes to middle lamella erosion and primary cell wall disintegration that results in softening and a decrease of firmness [20]. Cell wall hydrolytic enzymes such as *pectate lyase (PL), pectin acetylesterase (PAEs), polygalacturonase (PG),* and *β-galactosidase (β-gal)* are involved in the ripening process by solubilizing pectin polysaccharides of fruits [21]. In this study, the combined effect of pre-storage high-CO_2_ and 1-MCP treatment with CS reduced the rate of solubilization of pectin and PG activity. This could be attributed to the commonly downregulated genes that encode cell wall hydrolytic enzymes such as *endoglucanase 6, pectin acetylesterase 12, probable galacturonosyltransferase 15,* and *endo-1,3; 1,4-beta-D-glucanase* in both 1-MCP+CS and CO_2_+CS.

Expansins are thought to be involved in cell wall disassembly during fruit ripening and softening [22]. The genes encoding *expansin-A6* and *expansin-A4* were also downregulated in both 1-MCP+CS and CO_2_+CS, and could play a role in maintaining firmness of fruit as the cell walls do not disrupt for loosening and extension. The *probable pectate lyase 8* was also downregulated due to CO_2_+CS. Moreover, *vegetative cell wall protein gp1*, which is the major component of the outer cell wall [23], and the *probable aquaporin PIP2-5*, which facilitates the transport of water and solutes across cell membrane [24], were upregulated due to the 1-MCP+CS. Hence, supplementing CS with 1-MCP and high CO_2_ pre-storage treatment could reduce CI and maintain firmness of peach fruit, in turn prolonging the storage period.

The results of the present study also imply that storage at low temperature could be efficient in reducing respiration and ethylene production rates, but the weight loss was significantly hindered by pre-storage 1-MCP and CO_2_ treatment. The climacteric rise of respiration in fruits during ripening is caused by the ethylene and the final step of ethylene biosynthesis catalyzed by *1-aminocyclopropane-1-carboxylate oxidase (ACCO)* [21,25]. In this study, the downregulation of *1-aminocyclopropane-1-carboxylate oxidase homolog 1, transcript variant X1* in both 1-MCP and CO_2_ treatments could have been the possible reason for maintaining firmness and pectin content.

The ethylene signaling and response pathway includes ethylene response factors (ERFs), and ERFs act as a key regulatory hub in plant responses to abiotic stresses [26]. Moreover, *thaumatin-like protein 1* that could be involved in protecting tissues from pathogen infection [9], *transcription factor MYB1R1* [27], *zinc finger protein ZAT10* [28], and *bZIP transcription factor 60* [29] that expressed in response to environmental stresses were also upregulated in response to 1-MCP+CS and CO_2_+CS as compared to CS. Moreover, abiotic stress acclimation genes such as *cold shock protein CS66* [30] and *NAC transcription factor 29* [31] were upregulated due to 1-MCP+CS treatment. Hence, the upregulation of the above genes and transcription factors in the present study could be the response of the fruit to the stress caused by the exogenous 1-MCP and CO_2_ treatment.

*12-oxophytodienoate reductase 3 (OPR3)* is involved in the synthesis of jasmonic acid (JA) [32], which is known as a stress-related hormone [33]. In this study, *OPR3* was commonly downregulated due to 1-MCP+CS and CO_2_+CS treatments as compared to CS. In abiotic stress, JA is usually involved in physiological and molecular responses. Physiological responses include activation of antioxidant system and accumulation of amino acids and soluble sugars; the molecular responses involve the expression of JA-associated genes [33]. Moreover, the gene that encodes *glyceraldehyde-3-phosphate dehydrogenase (GAPDH)*, an essential component of the glycolytic pathway that converts glyceraldehyde-3-phosphate to 1,3-bisphosphoglycerate, was downregulated in this study due to 1-MCP+CS and CO_2_+CS treatments as compared to CS. In addition to its key role in glycolysis, *GAPDH* is involved in abiotic stress resistance in plants [34]. Taken together, delaying the synthesis of JA through downregulation of *OPR3* and downregulation of *GAPDH* could contribute to delaying of physiological and molecular responses, in turn prolonging the storage period of peach fruit treated with 1-MCP+CS and CO_2_+CS as compared to CS. 

The gene that encodes the glycosylation reaction for the accumulation of anthocyanin pigments, *anthocyanidin 3-O-glucosyltransferase 2 (3GT)* [35], and the gene that is involved in the transport and accumulation of anthocyanins and proanthocyanidins in the vacuole, *glutathione S-transferase F12 (GSTF12)* [36], were commonly downregulated due to 1-MCP+CS and CO_2_+CS. Moreover, the gene that encodes *3-hydroxy-3-methylglutaryl-coenzyme A reductase 1(HMGR)*, which catalyzes mevalonate pathway for isoprenoid (terpenoid) biosynthesis [37], was also downregulated due to 1-MCP+CS and CO_2_+CS. Hence, the downregulation of *3GT, GSTF12*, and *HMGR* due to 1-MCP+CS and CO_2_+CS could play in maintaining color of peach fruit as the treatments affect synthesis of the two groups of plant pigments, anthocyanins and carotenoids (tetraterpenoids) [38]. 

Wang and Luca [39] reported that *methanol O-anthraniloyltransferase (AMAT)* is an enzyme responsible for the production of *O-methyl anthranilate*, an important aroma and flavor compound in the grapefruit. Tilahun et al. [21] also confirmed the same in kiwifruit. In this study, the gene that encodes *AMAT* was commonly downregulated in peach fruit in response to 1-MCP+CS and CO_2_+CS. Similarly, the genes that encode *dihydroflavonol 4-reductase (DFR)* and *chalcone--flavonone isomerase (CHI)* were downregulated commonly in 1-MCP+CS and CO_2_+CS, and CO_2_+CS, respectively. *CHI* and *DFR* catalyze the conversion of chalcones into flavanones and dihydroflavonols into flavan-3,4-diols, respectively [40,41]. Hence, the downregulation of genes related to color changes and flavonoid biosynthesis could be an indicator of delayed ripening due to both 1-MCP+CS and CO_2_+CS as compared to CS fruit.

## 4. Materials and Methods

### 4.1. Plant Material and Treatments

For this experiment, fruit of ‘Madoka’ peach cultivar, one of the commonly grown cultivars around Chuncheon, Korea, was selected and harvested at commercial maturity on September 20, 2019. After harvesting, fruit were transported immediately to the postharvest laboratory at the Department of Horticultural Sciences, Kangwon National University. Uniform fruits free of defects were carefully selected and kept at 8 °C for 3 h to remove field heat, and then were treated with 0.5 µL L^−1^ 1-MCP (Smart Fresh, Agro fresh Korea Ltd., Seoul, Korea) for 24 h [42] and 30% CO_2_ for 6 h [10] in a sealed 62 L container at 0 °C [25]. Control fruit were treated at similar conditions without CO_2_ and 1-MCP treatment. Air in the sealed containers was ventilated and distributed by a fan (Coolertec CT8025L12RA-3P, Zhengzhou, China). Three containers were used for the treatments and control, and 30 fruit were placed in each container. The treated fruit were stored at 0 °C for 12 d (until the onset of chilling injury in the control fruit). Hence, the samples were categorized as fruit at commercial harvest (control), and three samples from the stored fruit on the 12 d (cold-stored (CS), 1-MCP+CS, and CO_2_+CS). Data for firmness, weight loss, respiration rate, ethylene production rate, and sensory evaluation were recorded at the beginning of the experiment and on the 12-d storage. Samples of fruit flesh were also taken for pectin content, polygalacturonase (PG) activity, and transcriptome analysis. The samples were frozen by liquid nitrogen and stored in a deep freezer (−80 °C) until analysis [21].

### 4.2. RNA Extraction and Sequencing Using Illumina Truseq Stranded mRNA Library Prep Kit

RNA extraction and sequencing were performed as described by Tilahun et al. [21] for three biological replicates from the control and treatments. Total RNA was extracted and pooled according to Park et al. [43]. RNA purity and integrity were checked as described by Johnson et al. [44], and mRNA sequencing was performed by the method followed by Kim [45]. 

### 4.3. Mapping Reads on a Reference Genome and Calculating Expression between Samples

Mapping and gene expression were performed according to Tilahun et al. [21]. Reads for each sample were mapped to the reference genome (*Prunus persica* (GCA_000346465.2)) by Tophat (v2.0.13) (http://ccb.jhu.edu/software/tophat/ accessed on 21 November 2020). The aligned results were then added to Cuffdiff (v2.2.0) (http://cole-trapnell-lab.github.io/cufflinks/cuffdiff/ accessed on 21 November 2020) to report differentially expressed genes. For library normalization and dispersion estimation, geometric and pooled methods (http://cole-trapnell-lab.github.io/cufflinks/cuffdiff/ accessed on 21 November 2020) were applied.

### 4.4. Identification of DEGs and Functional Enrichment Analysis

**Figure 6:**
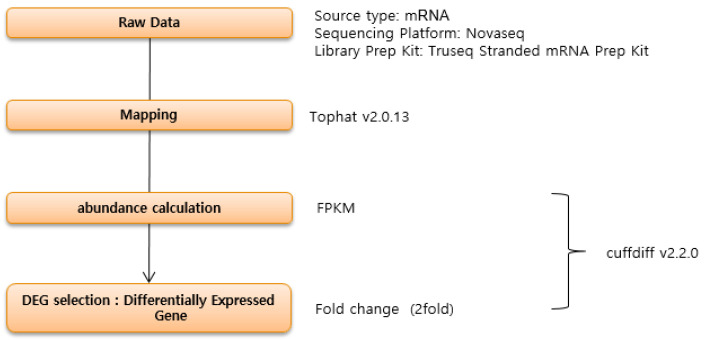
Flow chart of mRNA processing.

The method used by Kim [45] was implemented to detect DEGs between control and treatments after applying two filtering processes. Genes were filtered based on twofold change and *p*-value (*p* < 0.05), and DAVID (http://david.abcc.ncifcrf.gov/ accessed on 21 November 2020) was used to obtain a comprehensive set of functional annotations (Figure 6). 

### 4.5. Verification of DEGs by RT-qPCR

Transcript accumulation of *ERF113, ERF071, BAM3, TLP1, ZAT10, BZIP60, At1g64390, AMAT, EXPA6, EXPA4, PAE12,* and *DFR* was evaluated via quantitative real-time RCR (RT-PCR) as described by Park et al. [43] using gene-specific primers (Table 3). 

### 4.6. CI, Firmness, Pectin Content, and Polygalacturonase (PG) Activity

CI was assessed visually, and the CI index was calculated according to the method described by Lee [10]. Peach fruit firmness was measured by a Rheo meter (Sun Scientific Co. Ltd., Tokyo, Japan) from 10 fruit, two measurements per fruit, by a puncture at the equator with a maximum force of 10 kg and a 3 mm diameter round stainless steel probe with a flat end, as stated by Tilahun et al. [46]. PG activity and quantification of pectin were made following the methods described by Tilahun et al. [47]. 

### 4.7. Measurement of Weight loss, Respiration Rate, and Ethylene Production Rate

Fresh weight loss was measured as described by Tilahun et al. [48]. Peach fruit were weighed before storage and weighed again after 12-d storage to calculate percentage (%) weight loss during CS. Respiration rate and ethylene production rate of peach fruit was measured and expressed as described by Belew et al. [49]. 

### 4.8. Overall Sensory Evaluation

The overall acceptability of kiwifruit during the ripening period was evaluated as the mean value of the subjective scale made by 10 trained graduate students for flavor, color, sweetness, texture, and sponginess according to a subjective scale by Seo et al. [50] from bad (1) to excellent (5).

### 4.9. Statistical Analysis of Quality Parameters

Data of the considered quality parameters were expressed as means ± standard errors. SAS statistical software (SAS/STAT ^®^ 9.1; SAS Institute Inc., Cary, NC, USA) was implemented for statistical analyses, and Duncan’s multiple range test was performed to observe differences between the treatment means.

## 5. Conclusions

The transcriptome analysis of ‘Madoka’ peach fruit treated with 1-MCP and high CO_2_ and stored for 12 d at 0 °C is reported in this study. To study the candidate genes linked to CI and cold storage of peach fruit, we compared the treated fruit with the control fruit. Several genes were differentially expressed in 1-MCP+CS- and CO_2_+CS-treated fruits as compared to CS. We also identified the list of genes that were expressed exclusively and commonly in the fruit treated by 1-MCP+CS and CO_2_+CS and compared them to the fruit quality parameters to study the changes observed after treatment. The findings showed that the effect of pre-storage treatments inhibited CI and delayed the process of ripening, which may lead to a longer storage period. An attempt was also made to identify and categorize genes related to softening, physiological changes, and other ripening-related changes. This study adds information on the molecular mechanisms of the pre-storage treatments during cold storage of peach fruit. Understanding the genetic response of susceptible cultivars uch as ‘Madoka’ to CI-reducing pre-storage treatments would help breeders release CI-resistant cultivars and could help postharvest technologists to develop more CI- reducing technologies. 

## Figures and Tables

**Table 1:** Summary of the mapping rate at which readings for each peach sample are mapped to the reference genome.

Sample Name	Total Reads	Mapped Reads	Mapping Rate
Control	44,051,176	41,516,721	94.25
CS	62,460,608	59,571,061	95.37
1-MCP+CS	60,048,660	58,007,008	96.60
CO_2_+CS	62,411,254	59,837,852	95.88
Average	57,242,925	54,733,161	95.53

**Table 2:** Summary of the average sequencing result.

Sample Name	Total Bases	Q30 Bases	Read Count	% ≥Q30 Bases	Mean Quality Score (PF)
Control	2,492,398,917	2,350,084,087	24,677,217	94.29	35.94
CS	3,575,251,126	3,410,220,666	35,398,526	95.38	36.20
1-MCP+CS	3,269,381,110	3,135,881,737	32,370,110	95.91	36.31
CO_2_+CS	3,592,272,151	3,442,927,010	35,567,051	95.84	36.29

**Table 3:** Selected genes and primers used for validation of the transcriptome analysis with the quantitative RT-PCR.

Gene_Id	Gene Descriptions	Primer Sequence 5’–3’-Forward	Reverse
LOC18790383	*ethylene-responsive transcription factor ERF113 (ERF113)*	GGCTAGTGCATCTCCTCATTAC	CAGTGCCTGGCTTCGATAAA
LOC18781898	*thaumatin-like protein 1 (TLP1)*	GGGATCTGATGGAAGCGTAAT	GTCTCCGGCTTGTCGTTAG
LOC18777635	*beta-amylase 3, chloroplastic (BAM3)*	ACTCATGCAGCATTCCTCTAC	GGATTCCTCCTGCCTGATTT
LOC18767585	*ethylene-responsive transcription factor ERF071 (ERF071)*	TGGGATTCACTGGCACTATG	GTTGGTAGGTAACCGTCTCTATG
LOC18792372	*zinc finger protein ZAT10 (ZAT10)*	CGAGACCTTTGACCTGAACAT	CGTCAATATCCTGGGCTTCTT
LOC18776076	*bZIP transcription factor 60 (BZIP60)*	CGTTGCTCTGCCTCTAATTCT	CTCTTGGCCTTAGATCCACATT
LOC18776275	*endoglucanase 6 (At1g64390)*	CTGCAAGTGGTGAGCTTAGT	GTAGTGGTCAGTGTTCCCATC
LOC18774729	*expansin-A6 (EXPA6)*	CGAGTACAGAGCTGGAATTGT	AGTAACGGAAGCCGTTGATAG
LOC18773967	*expansin-A4 (EXPA4)*	CTCCTCTCCAGCACTTTGATT	TTCTTCATACAGGGCACTCTTC
LOC18785045	*anthocyanidin 3-O-glucosyltransferase 2 (3GT)*	CGCCTCACCTGCTTGATTA	ATTAAGTCCGGAGAGCCAAAG
LOC18775995	*methanol O-anthraniloyltransferase (AMAT)*	CCCAAGGAGCAGATTCACTATC	TCGTAATGCCAACGTGTAACT
LOC18775065	*pectin acetylesterase 12 (PAE12)*	TGGTGGGTTGGCATTGTAATA	TCTCCATAGGCCTCCAAGAA
LOC18782821	*glutathione S-transferase F12 (GSTF12)*	CCAGCAGTAGAAGATGGTGATT	CTCCAGGGTTGTTCCCAATAG
LOC18788884	*dihydroflavonol 4-reductase (DFR)*	CGAAGAGCACCAGAAGTCATAC	CTAGAGTCTTGGAGGCGAAGTA
LOC18789799	*1-aminocyclopropane-1-carboxylate oxidase (ACO1)*	GGAGACCAACTCTTCGGATTG	GGATAGTAGTGGCACACAAAGG

## Data Availability

All data sets are available upon reasonable request from the corresponding author.

## Supplementary Materials

The following are available online at https://www.mdpi.com/article/10.3390/ijms22094437/s1. Table S1: List of commonly expressed and identified DEGs in the comparison of CS vs. CO_2_ + CS and 1-MCP + CS ‘Madoka’ peach fruit after 12 days storage. Table S2: List of exclusively expressed and identified DEGs in the comparison of control vs. CS with control vs. 1-MCP + CS ‘Madoka’ peach fruit after 12 days storage. Table S3: List of exclusively expressed and identified DEGs in the comparison of control vs. CS with control vs. CO_2_ + CS ‘Madoka’ peach fruit after 12 days storage.
