# Supplementary material for: Transcriptome Analysis of Pre-Storage 1-MCP and High CO2-Treated ‘Madoka’ Peach Fruit Explains the Reduction in Chilling Injury and Improvement of Storage Period by Delaying Ripening
Source: Int J Mol Sci. 2021 Apr 23;22(9):4437. doi: 10.3390/ijms22094437 (PMC8123058; doi:10.3390/ijms22094437)
Supplement: Supplementary file 1 [file ijms-22-04437-s001.zip › ijms-1125524-supplementary.pdf]

International Journal of *Molecular Sciences*

MDPI

Supplementary Material

Transcriptome analysis of prestorage 1-MCP and high CO_2 treated 'Madoka' peach fruit explains the reduction in chilling injury and improvement of storage period by delaying ripening

Han Ryul Choi[1,2], Min Jae Jeong[1,2], Min Woo Baek[1,2], Jong Hang Choi[1], Hee Cheol Lee[1,2], Cheon Soon Jeong[1,2,*], Shimeles Tilahun[1,3,4,*]

[1] Department of Horticulture, Kangwon National University, Chuncheon 24341, Korea; hanryul192@kangwon.ac.kr (H.R.C.); jmj717@naver.com (M.J.J.); minwoo100@kangwon.ac.kr (M.W.B.);choijh107@kangwon.ac.kr (J.H.C.); yaya9459@kangwon.ac.kr (H.C.L.); jeongcs@kangwon.ac.kr (C.S.J.); shimeles@kangwon.ac.kr (S.T.)

[2] Interdisciplinary Program in Smart Agriculture, Kangwon National University, Chuncheon 24341, Korea

[3] Agriculture and Life Science Research Institute, Kangwon National University, Chuncheon 24341, Korea

[4] Department of Horticulture and Plant Sciences, Jimma University, Jimma 378, Ethiopia

* Correspondence: Correspondence: jeongcs@kangwon.ac.kr; shimeles@kangwon.ac.kr; Tel.: +82-033-250-6409

International Journal of
Molecular Sciences

Table S1. List of commonly expressed and identified DEGs in the comparison of CS vs. CO_2 + CS and 1-MCP + CS 'Madoka' peach fruit after 12 days storage. 1

Gene_id	mRNA_accession	Protein accession	Gene descriptions	Log fold change		*p* value	
				CO_2	1-MCP	CO_2	1-MCP
**Up-regulated**							
LOC18790383	XM_020555209.1	XP_020410798.1	*ethylene-responsive transcription factor ERF113*	*6.81*	*6.62*	*0.042*	*0.042*
LOC18781898	XM_007215803.2	XP_007215865.1	*thaumatin-like protein 1*	*6.73*	*8.94*	*0.034*	*0.033*
LOC18790734	XM_007222992.2	XP_007223054.2	*esterase*	*6.67*	*6.62*	*0.039*	*0.038*
LOC18777635	XM_007209805.2	XP_007209867.1	*beta-amylase 3, chloroplastic*	*6.48*	*6.13*	*0.001*	*0.001*
LOC18788559	XM_007225606.2	XP_007225668.1	*putative beta-D-xylosidase*	*6.32*	*5.20*	*0.001*	*0.001*
LOC18767585	XM_007200356.2	XP_007200418.1	*ethylene-responsive transcription factor ERF071*	*6.26*	*7.52*	*0.001*	*0.001*
LOC18766816	XM_007201832.2	XP_007201894.1	*expansin-like A2*	*5.78*	*6.46*	*0.001*	*0.001*
LOC18776099	XM_007210904.2	XP_007210966.2	*E3 ubiquitin-protein ligase BOI, transcript variant X1*	*5.73*	*6.10*	*0.001*	*0.001*
LOC18789693	XM_007222580.2	XP_007222642.1	*transcription factor MYB1R1*	*5.57*	*6.17*	*0.001*	*0.001*
LOC18792372	XM_007222812.2	XP_007222874.1	*zinc finger protein ZAT10*	*5.36*	*6.02*	*0.001*	*0.007*
LOC18789880	XM_007222978.2	XP_007223040.1	*probable galacturonosyltransferase-like 9*	*4.85*	*5.57*	*0.003*	*0.002*
LOC18790715	XM_007222877.2	XP_007222939.1	*lipase*	*4.58*	*4.44*	*0.001*	*0.001*
LOC18781566	XM_007215176.2	XP_007215238.1	*gamma aminobutyrate transaminase 3, chloroplastic*	*4.46*	*3.64*	*0.023*	*0.006*
LOC18767804	XM_007199131.2	XP_007199193.2	*bifunctional riboflavin biosynthesis protein RIBA 1, chloroplastic*	*4.23*	*4.52*	*0.001*	*0.001*
LOC18768297	XM_007200926.2	XP_007200988.1	*scopoletin glucosyltransferase*	*4.13*	*4.78*	*0.002*	*0.001*
LOC18783318	XM_007214979.2	XP_007215041.1	*allene oxide cyclase, chloroplastic*	*3.93*	*5.10*	*0.002*	*0.001*
LOC18768213	XM_020570312.1	XP_020425901.1	*pleiotropic drug resistance protein 1*	*3.78*	*4.53*	*0.001*	*0.001*
LOC18791409	XM_007222632.2	XP_007222694.1	*E3 ubiquitin-protein ligase ATL6*	*3.72*	*5.12*	*0.001*	*0.001*
LOC18773198	XM_007205323.2	XP_007205385.1	*omega-3 fatty acid desaturase, chloroplastic*	*3.71*	*3.49*	*0.001*	*0.002*
LOC18793580	*XM_007227653.2*	XP_007227715.1	*transcription factor MYB44*	*3.67*	*4.31*	*0.014*	*0.045*
LOC18767634	XM_007199720.2	XP_007199782.1	*lysine histidine transporter-like 8*	*3.62*	*4.73*	*0.017*	*0.004*
LOC18789531	XM_007222768.2	XP_007222830.1	*cinnamoyl-CoA reductase-like SNL6, transcript variant X3*	*3.36*	*3.83*	*0.043*	*0.031*
LOC18789736	XM_007223025.2	XP_007223087.1	*jasmonate O-methyltransferase*	*3.26*	*3.48*	*0.005*	*0.004*
LOC18776076	XM_007209268.2	XP_007209330.1	*bZIP transcription factor 60*	*3.06*	*4.40*	*0.026*	*0.027*

Cont'd

Gene_id	mRNA_accession	Protein accession	Gene descriptions	Log fold change		*p* value	
				CO_2	1-MCP	CO_2	1-MCP
LOC18771212	XM_007204294.2	XP_007204356.2	*glucan endo-1,3-beta-glucosidase 14*	*3.01*	*2.16*	*0.007*	*0.049*
LOC18789209	XM_007223513.2	XP_007223575.1	*cell wall / vacuolar inhibitor of fructosidase 1*	*2.90*	*4.31*	*0.006*	*0.001*
LOC109946619	XM_020555005.1	XP_020410594.1	*endoglucanase 10*	*2.90*	*3.17*	*0.003*	*0.001*
LOC18773658	XM_020565001.1	XP_020420590.1	*AP2-likeethylene-responsive transcription factor TOE3, transcript variant X2*	*2.88*	*3.37*	*0.042*	*0.026*
LOC18774914	XM_007205621.2	XP_007205683.1	*methylesterase 17*	*2.83*	*2.89*	*0.009*	*0.008*
LOC18768526	XM_007200507.2	XP_007200569.1	*E3 ubiquitin-protein ligase CHIP*	*2.45*	*2.94*	*0.009*	*0.012*
LOC18775963	XM_007209024.2	XP_007209086.1	*4-coumarate--CoA ligase-like 9*	*2.16*	*3.13*	*0.026*	*0.006*
LOC18786692	XM_007218108.2	XP_007218170.1	*glycosyltransferase family 64 protein C4*	*2.01*	*2.40*	*0.041*	*0.013*
**Down-regulated**							
LOC18776275	XM_007210219.2	XP_007210281.1	*endoglucanase 6*	*-7.12*	*-5.09*	*0.001*	*0.001*
LOC18774729	XM_007205678.2	XP_007205740.1	*expansin-A6*	*-6.04*	*-4.03*	*0.001*	*0.001*
LOC18770721	XM_020567830.1	XP_020423419.1	*beta-galactosidase 3*	*-5.69*	*-4.96*	*0.040*	*0.002*
LOC18771540	XM_007204321.2	XP_007204383.1	*12-oxophytodienoate reductase 3*	*-5.24*	*-3.58*	*0.001*	*0.004*
LOC18773967	XM_007205700.2	XP_007205762.1	*expansin-A4*	*-5.08*	*-3.46*	*0.001*	*0.001*
LOC18785045	XM_007217890.2	XP_007217952.1	*anthocyanidin 3-O-glucosyltransferase 2*	*-4.92*	*-4.60*	*0.001*	*0.001*
LOC18775995	XM_007209069.2	XP_007209131.1	*methanol O-anthraniloyltransferase*	*-4.66*	*-3.29*	*0.001*	*0.003*
LOC18774870	XM_007208112.2	XP_007208174.1	*probable linoleate 9S-lipoxygenase 5*	*-4.44*	*-4.32*	*0.001*	*0.001*
LOC18784809	XM_007220569.2	XP_007220631.1	*jasmonic acid-amido synthetase JAR1, transcript variant X2*	*-4.33*	*-3.27*	*0.002*	*0.010*
LOC18775065	XM_020566833.1	XP_020422422.1	*pectin acetylesterase 12*	*-4.17*	*-5.51*	*0.001*	*0.001*
LOC18786085	XM_007218621.2	XP_007218683.1	*cinnamoyl-CoA reductase 1*	*-4.00*	*-4.51*	*0.006*	*0.003*
LOC18783897	XM_007215328.2	XP_007215390.2	*probable galacturonosyltransferase 15*	*-3.68*	*-3.21*	*0.001*	*0.003*
LOC18782821	XM_007215891.2	XP_007215953.1	*glutathione S-transferase F12*	*-3.66*	*-4.09*	*0.001*	*0.001*
LOC18770980	XM_007204516.2	XP_007204578.1	*3-hydroxy-3-methylglutaryl-coenzyme A reductase 1*	*-3.63*	*-2.92*	*0.002*	*0.003*
LOC18788884	XM_007222255.2	XP_007222317.1	*dihydroflavonol 4-reductase*	*-3.56*	*-3.46*	*0.002*	*0.004*

Cont'd

**Gene_id**	**mRNA_accession**	**Protein accession**	**Gene descriptions**	**Log fold change**		***p* value**	
				**CO_2**	**1-MCP**	**CO_2**	**1-MCP**
LOC18783067	XM_007215424.2	XP_007215486.1	*ATP-dependent 6-phosphofructokinase 6*	-3.35	-2.96	0.001	0.005
LOC18789799	XM_020555946.1	XP_020411535.1	*1-aminocyclopropane-1-carboxylate oxidase homolog 1, transcript variant X1*	-3.19	-3.36	0.024	0.002
LOC18769691	XM_007204670.2	XP_007204732.1	*soluble inorganic pyrophosphatase*	-3.15	-3.36	0.010	0.004
LOC18781238	XM_007214493.2	XP_007214555.2	*glycogen phosphorylase 1*	-3.14	-3.02	0.006	0.007
LOC18778082	*XM_007210461.2*	XP_007210523.1	*glyceraldehyde-3-phosphate dehydrogenase, cytosolic*	-3.06	-2.47	0.015	0.044
LOC18779670	*XM_007211818.2*	XP_007211880.1	*pyrophosphate--fructose 6-phosphate 1-phosphotransferase subunit beta*	-3.03	-2.75	0.004	0.010
LOC18781433	XM_007214656.2	XP_007214718.1	*glyceraldehyde-3-phosphate dehydrogenase GAPCP1, chloroplastic*	-2.71	-2.13	0.011	0.047
LOC18783403	XM_007215938.2	XP_007216000.1	*glucose-1-phosphate adenylyltransferase small subunit, chloroplastic/amyloplastic*	-2.55	-2.33	0.016	0.028
LOC18769676	XM_007204579.2	XP_007204641.1	*subtilisin-like protease SBT3.5*	-2.52	-3.44	0.009	0.003
LOC18793152	XM_007223728.2	XP_007223790.1	*endo-1,3;1,4-beta-D-glucanase*	-2.35	-2.08	0.015	0.029
LOC18781318	XM_007211928.2	XP_007211990.1	*DAG protein, chloroplastic*	-2.28	-2.42	0.017	0.032
LOC18789462	XM_007225326.2	XP_007225388.1	*calcium-transporting ATPase 1, chloroplastic*	-2.09	-2.00	0.038	0.048
LOC18791377	XM_020554552.1	XP_020410141.1	*phosphoenolpyruvate carboxylase 4*	-2.09	-2.17	0.049	0.043

Table S2. List of exclusively expressed and identified DEGs in the comparison of control vs. CS with control vs. 1-MCP+ CS 'Madoka' peach fruit after 12 days storage.

Gene_id	mRNA accession	Protein accession	Gene descriptions	Log fold change	*p* value
**Up-regulated**					
LOC18771354	XM_007202534.2	XP_007202596.2	*cold shock protein CS66*	4.52	0.000
LOC18792096	XM_007223262.2	XP_007223324.1	*NAC transcription factor 29*	4.42	0.049
LOC18774119	XM_007205943.2	XP_007206005.1	*vegetative cell wall protein gp1*	3.84	0.009
LOC18772963	XM_007207282.2	XP_007207344.1	*probable aquaporin PIP2-5*	3.71	0.008
LOC18781808	XM_007215851.2	XP_007215913.1	*26.5 kDa heat shock protein, mitochondrial*	3.62	0.015
LOC18771504	XM_007203666.2	XP_007203728.1	*UDP-glycosyltransferase 73C3*	3.58	0.010
LOC18783057	XM_007217136.2	XP_007217198.1	*non-specific lipid-transfer protein 2*	3.28	0.003
LOC18784147	XM_007215806.2	XP_007215868.1	*B-box zinc finger protein 24*	3.28	0.005
LOC18766758	XM_007201292.2	XP_007201354.1	*NDR1/HIN1-like protein 13*	3.22	0.003
LOC18782396	XM_007217705.2	XP_007217767.1	*zinc finger AN1 domain-containing stress-associated protein 12*	3.18	0.019
LOC18768241	XM_007201304.2	XP_007201366.1	*peroxidase 12*	3.11	0.010
LOC18770453	XM_007203623.2	XP_007203685.1	*protein EARLY-RESPONSIVE TO DEHYDRATION 7, chloroplastic*	3.09	0.006
LOC18769499	XM_007202413.2	XP_007202475.1	*aquaporin TIP1-1*	3.07	0.042
LOC18772208	XM_007205406.2	XP_007205468.2	*F-box/kelch-repeat protein At1g15670*	3.07	0.005
LOC18769333	XM_007202197.2	XP_007202259.1	*fructose-bisphosphate aldolase, cytoplasmic isozyme*	3.02	0.003
LOC18781387	XM_007211912.2	XP_007211974.1	*polyphenol oxidase, chloroplastic*	3.00	0.031
LOC18784924	XM_007218708.2	XP_007218770.1	*protein SRC2*	2.95	0.011
LOC18779840	XM_020563111.1	XP_020418700.1	*VQ motif-containing protein 4*	2.94	0.029
LOC18773341	XM_007207361.2	XP_007207423.1	*UDP-glycosyltransferase 71K1, transcript variant X1*	2.90	0.033
LOC18766770	XM_007201642.2	XP_007201704.2	*chaperonin CPN60-2, mitochondrial*	2.86	0.004
LOC18785553	XM_007217962.2	XP_007218024.1	*UDP-glucuronate 4-epimerase 3*	2.79	0.009
LOC18783598	XM_007217098.2	XP_007217160.1	*homeobox-leucine zipper protein GLABRA 2*	2.75	0.014
LOC18766694	XM_020569952.1	XP_020425541.1	*BTB/POZ domain-containing protein At1g63850*	2.72	0.009

Cont'd

**Gene_id**	**mRNA accession**	**Protein accession**	**Gene descriptions**	**Log fold change**	***p* value**
LOC18780582	XM_007211964.2	XP_007212026.1	*probable polygalacturonase*	*2.71*	*0.016*
LOC18785140	XM_007218771.2	XP_007218833.1	*ethylene-responsive transcription factor 2*	*2.69*	*0.016*
LOC18782289	XM_007215565.2	XP_007215627.1	*probable 2-oxoglutarate-dependent dioxygenase At5g05600*	*2.68*	*0.013*
LOC18772792	XM_020566162.1	XP_020421751.1	*glycine-rich RNA-binding protein RZ1A*	*2.67*	*0.016*
LOC18785398	XM_020556175.1	XP_020411764.1	*probable serine/threonine-protein kinase PBL9*	*2.63*	*0.007*
LOC18774023	XM_020566630.1	XP_020422219.1	*ethylene-responsive transcription factor RAP2-7, transcript variant X1*	*2.60*	*0.046*
LOC18767475	XM_020569665.1	XP_020425254.1	*magnesium transporter MRS2-3*	*2.58*	*0.022*
LOC18770251	XM_007202669.2	XP_007202731.1	*multiprotein-bridging factor 1c*	*2.57*	*0.019*
LOC18786863	XM_007218228.2	XP_007218290.1	*enoyl-CoA delta isomerase 1, peroxisomal*	*2.55*	*0.020*
LOC18772307	XM_007205984.2	XP_007206046.1	*zinc finger A20 and AN1 domain-containing stress-associated protein 3*	*2.55*	*0.014*
LOC18766507	XM_007199633.2	XP_007199695.2	*calcium-transporting ATPase 12, plasma membrane-type*	*2.54*	*0.023*
LOC18787090	XM_007218734.2	XP_007218796.1	*glycine-rich RNA-binding protein RZ1C, transcript variant X1*	*2.53*	*0.016*
LOC18769680	XM_007204563.2	XP_007204625.1	*wall-associated receptor kinase-like 14*	*2.52*	*0.010*
LOC18780279	XM_007211916.2	XP_007211978.1	*ethylene-responsive transcription factor 3*	*2.52*	*0.015*
LOC18767780	XM_007200991.2	XP_007201053.1	*alcohol dehydrogenase 1*	*2.51*	*0.024*
LOC18767388	XM_007201305.2	XP_007201367.1	*cinnamyl alcohol dehydrogenase 1*	*2.45*	*0.026*
LOC18770307	XM_020568329.1	XP_020423918.1	*polygalacturonase ADPG2*	*2.44*	*0.011*
LOC18778952	XM_007213821.2	XP_007213883.1	*bifunctional riboflavin kinase/FMN phosphatase*	*2.43*	*0.030*
LOC18786375	XM_007220153.2	XP_007220215.1	*L-type lectin-domain containing receptor kinase S.4*	*2.43*	*0.029*
LOC18773417	XM_020565931.1	XP_020421520.1	*4-alpha-glucanotransferase, chloroplastic/amyloplastic, transcript variant X2*	*2.42*	*0.012*
LOC18768018	XM_007200293.2	XP_007200355.1	*UDP-galactose transporter 2*	*2.42*	*0.012*
LOC18781646	XM_007215020.2	XP_007215082.1	*17.4 kDa class III heat shock protein*	*2.40*	*0.036*
LOC18766098	XM_007198776.2	XP_007198838.1	*E3 ubiquitin-protein ligase RMA1H1*	*2.37*	*0.035*
LOC18772229	XM_020566279.1	XP_020421868.1	*alkaline/neutral invertase E, chloroplastic, transcript variant X2*	*2.34*	*0.033*
LOC109946804	XM_020555654.1	XP_020411243.1	*glycine-rich protein 2-like*	*2.34*	*0.036*

Cont'd

**Gene_id**	**mRNA accession**	**Protein accession**	**Gene descriptions**	**Log fold change**	***p* value**
LOC18791585	XM_020569234.1	XP_020424823.1	*probable protein phosphatase 2C 59*	*2.34*	*0.030*
LOC18770835	XM_007202017.2	XP_007202079.1	*phosphoserine aminotransferase 1, chloroplastic*	*2.30*	*0.030*
LOC18785040	XM_020558370.1	XP_020413959.1	*L-type lectin-domain containing receptor kinase VIII.2*	*2.29*	*0.032*
LOC18768133	XM_020569850.1	XP_020425439.1	*patellin-6*	*2.28*	*0.037*
LOC18777090	XM_007209028.2	XP_007209090.1	*beta-amylase 3, chloroplastic*	*2.26*	*0.032*
LOC18768033	XM_007201220.2	XP_007201282.1	*universal stress protein PHOS34*	*2.21*	*0.037*
LOC18776383	XM_007209540.2	XP_007209602.1	*heat shock 22 kDa protein, mitochondrial*	*2.21*	*0.042*
LOC109946242	XM_020565479.1	XP_020421068.1	*probable histone H2B.1*	*2.21*	*0.022*
LOC18769662	XM_007202310.2	XP_007202372.1	*heat stress transcription factor B-1*	*2.20*	*0.042*
LOC18789842	XM_007222319.2	XP_007222381.1	*UDP-glucuronate 4-epimerase 1*	*2.19*	*0.037*
LOC18767607	XM_007201272.2	XP_007201334.1	*SKP1-interacting partner 15*	*2.13*	*0.045*
LOC18769952	XM_007202265.2	XP_007202327.1	*F-box protein SKIP1*	*2.12*	*0.047*
LOC18774456	XM_007205974.2	XP_007206036.1	*60S ribosomal protein L12*	*2.10*	*0.029*
LOC18784867	XM_007220189.2	XP_007220251.1	*golgin candidate 4*	*2.08*	*0.036*
LOC18767471	XM_007200989.2	XP_007201051.1	*BTB/POZ and MATH domain-containing protein 2*	*2.02*	*0.034*
LOC18784947	XM_007220253.2	XP_007220315.2	*glucan endo-1,3-beta-glucosidase*	*2.02*	*0.039*
LOC18788282	XM_007225331.2	XP_007225393.1	*putative calcium-transporting ATPase 13, plasma membrane-type*	*2.02*	*0.034*
LOC18785734	XM_007218126.2	XP_007218188.1	*F-box/kelch-repeat protein SKIP4*	*2.01*	*0.035*
LOC18786278	XM_020557544.1	XP_020413133.1	*protein TRANSPARENT TESTA GLABRA 1, transcript variant X1*	*2.01*	*0.039*
LOC18767346	XM_007200378.2	XP_007200440.2	*epoxide hydrolase A*	*2.00*	*0.043*
LOC18766155	XM_007201165.2	XP_007201227.1	*RPM1-interacting protein 4*	*2.00*	*0.047*
**Down-regulated**					
LOC18783695	XM_007215345.2	XP_007215407.1	*phytoene synthase 2, chloroplastic*	*-4.58*	*0.003*
LOC18782475	XM_020559547.1	XP_020415136.1	*ABC transporter I family member 11, chloroplastic, transcript variant X2*	*-4.09*	*0.029*
LOC18785488	XM_007218726.2	XP_007218788.1	*phosphoglucan phosphatase LSF2, chloroplastic*	*-3.72*	*0.008*

Cont'd

Gene_id	mRNA accession	Protein accession	Gene descriptions	Log fold change	*p* value
LOC18790537	XM_007222967.2	XP_007223029.1	*caffeoylshikimate esterase*	-3.64	0.009
LOC18768052	XM_020570437.1	XP_020426026.1	*probable ethylene response sensor 1, transcript variant X2*	-3.37	0.006
LOC18771959	XM_007208512.2	XP_007208574.2	*fasciclin-like arabinogalactan protein 4*	-3.36	0.016
LOC18783832	XM_007217106.2	XP_007217168.1	*phosphoglucan phosphatase LSF1, chloroplastic, transcript variant X1*	-3.36	0.017
LOC18769772	XM_020567700.1	XP_020423289.1	*B3 domain-containing transcription factor VRN1, transcript variant X2*	-3.30	0.037
LOC109946232	XM_007209634.2	XP_007209696.1	*probable phospholipid hydroperoxide glutathione peroxidase*	-2.84	0.011
LOC18776292	XM_020563447.1	XP_020419036.1	*methanol O-anthraniloyltransferase*	-2.77	0.039
LOC18784624	XM_007220161.2	XP_007220223.1	*beta-amylase 7*	-2.71	0.044
LOC18771811	XM_007201984.2	XP_007202046.1	*lycopene beta cyclase, chloroplastic/chromoplastic*	-2.70	0.009
LOC18781009	XM_007211705.2	XP_007211767.1	*ycf3-interacting protein 1, chloroplastic*	-2.70	0.046
LOC18770198	XM_020568695.1	XP_020424284.1	*cold-regulated 413 plasma membrane protein 2, transcript variant X2*	-2.65	0.046
LOC18776549	XM_020564416.1	XP_020420005.1	*ethylene-responsive transcription factor ERF118, transcript variant X1*	-2.65	0.047
LOC18777969	XM_007209427.2	XP_007209489.1	*bidirectional sugar transporter SWEET4*	-2.56	0.020
LOC18786335	XM_007218842.2	XP_007218904.1	*beta-galactosidase*	-2.52	0.008
LOC18769184	XM_007203569.2	XP_007203631.1	*DELLA protein DWARF8*	-2.49	0.024
LOC18786577	XR_002270240.1		*pyruvate kinase 1, cytosolic*	-2.46	0.033
LOC18767724	XM_007201691.2	XP_007201753.1	*ETO1-like protein 1, transcript variant X1*	-2.34	0.038
LOC18782183	XM_007215315.2	XP_007215377.1	*protein farnesyltransferase subunit beta*	-2.24	0.043
LOC18786310	XM_007220464.2	XP_007220526.1	*NADP-dependent D-sorbitol-6-phosphate dehydrogenase*	-2.16	0.045
LOC18785044	XM_007218342.2	XP_007218404.1	*soluble inorganic pyrophosphatase 1*	-2.16	0.046
LOC18766941	XM_007201311.2	XP_007201373.1	*probable 2-oxoglutarate-dependent dioxygenase At5g05600*	-2.11	0.049
LOC18779016	XM_020561850.1	XP_020417439.1	*starch synthase 3, chloroplastic/amyloplastic, transcript variant X2*	-2.01	0.039
LOC18781818	XM_007215454.2	XP_007215516.1	*pyrophosphate--fructose 6-phosphate 1-phosphotransferase subunit beta*	-2.01	0.040

Table S3. List of exclusively expressed and identified DEGs in the comparison of control vs. CS with control vs. CO_2 + CS 'Madoka' peach fruit after 12 days storage.

Gene_id	mRNA_accession	Protein accession	Gene descriptions	Log fold change	*p* value
**Up-regulated**					
LOC18777938	XM_007210330.2	XP_007210392.1	*disease resistance protein RPM1*	*5.00*	*0.047*
LOC18778873	XM_020562922.1	XP_020418511.1	*E3 ubiquitin-protein ligase RMA1H1*	*4.29*	*0.006*
LOC109949277	XM_020564500.1	XP_020420089.1	*zinc finger MYM-type protein 1-like, transcript variant X1*	*3.79*	*0.046*
LOC18792502	XM_007223052.2	XP_007223114.2	*beta-amylase 1, chloroplastic*	*3.54*	*0.038*
LOC109946750	XM_020555453.1	XP_020411042.1	*MDIS1-interacting receptor like kinase 2-like*	*3.51*	*0.015*
LOC18781808	XM_007215851.2	XP_007215913.1	*26.5 kDa heat shock protein, mitochondrial*	*3.38*	*0.020*
LOC18785140	XM_007218771.2	XP_007218833.1	*ethylene-responsive transcription factor 2*	*3.33*	*0.005*
LOC18792321	XM_007222045.2	XP_007222107.1	*probable xyloglucan endotransglucosylase /hydrolase protein 23*	*3.32*	*0.048*
LOC18784769	XM_007218307.2	XP_007218369.1	*homeobox-leucine zipper protein ATHB-12*	*3.27*	*0.005*
LOC18782384	XM_007215690.2	XP_007215752.1	*Xyloglucan endotransglucosylase/hydrolase protein 9*	*3.22*	*0.008*
LOC18781249	XM_007213862.2	XP_007213924.2	*non-specific phospholipase C2*	*3.18*	*0.024*
LOC18792450	XM_020554466.1	XP_020410055.1	*putative disease resistance protein At1g50180, transcript variant X2*	*3.14*	*0.015*
LOC18786254	XM_007220340.2	XP_007220402.1	*probable protein phosphatase 2C 25*	*2.99*	*0.033*
LOC109950746	XM_020570777.1	XP_020426366.1	*phosphopantothenoylcysteine decarboxylase subunit VHS3*	*2.92*	*0.041*
LOC109948313	XM_020560949.1	XP_020416538.1	*prostatic spermine-binding protein-like*	*2.90*	*0.038*
LOC18766151	XM_020553614.1	XP_020409203.1	*CCG-binding protein 1*	*2.88*	*0.006*
LOC18782080	XM_007214780.2	XP_007214842.1	*chaperone protein dnaJ 8, chloroplastic*	*2.79*	*0.009*
LOC109946468	XM_020554385.1	XP_020409974.1	*G-type lectin S-receptor-like serine/threonine-protein kinase SD2-5*	*2.79*	*0.039*
LOC18783132	XM_020559174.1	XP_020414763.1	*Probable receptor-like serine/threonine-protein kinase LRK10L-1.4*	*2.78*	*0.021*
LOC18767054	XM_007200310.2	XP_007200372.1	*nitronate monooxygenase*	*2.71*	*0.046*
LOC18766098	XM_007198776.2	XP_007198838.1	*E3 ubiquitin-protein ligase RMA1H1*	*2.65*	*0.017*
LOC18785778	XM_007219774.2	XP_007219836.2	*glycerol-3-phosphate dehydrogenase [NAD(+)] 2, chloroplastic*	*2.61*	*0.047*
LOC18772963	XM_007207282.2	XP_007207344.1	*probable aquaporin PIP2-5*	*2.58*	*0.047*

Cont'd

Gene_id	mRNA_accession	Protein accession	Gene descriptions	Log fold change	*p* value
LOC18785553	XM_007217962.2	XP_007218024.1	*UDP-glucuronate 4-epimerase 3*	*2.46*	*0.021*
LOC18766119	XM_007198838.2	XP_007198900.1	*transcriptional regulator DEF1, transcript variant X2*	*2.46*	*0.016*
LOC18791459	XM_020562849.1	XP_020418438.1	*disease resistance protein RGA2, transcript variant X1*	*2.45*	*0.013*
LOC18770307	XM_020568329.1	XP_020423918.1	*polygalacturonase ADPG2*	*2.44*	*0.011*
LOC18780582	XM_007211964.2	XP_007212026.1	*probable polygalacturonase*	*2.36*	*0.032*
LOC18772623	XM_007208228.2	XP_007208290.1	*WRKY transcription factor WRKY24*	*2.34*	*0.036*
LOC18766055	XM_007198843.2	XP_007198905.2	*callose synthase 12*	*2.29*	*0.019*
LOC18776744	XM_007210319.2	XP_007210381.1	*chaperone protein ClpB1*	*2.28*	*0.019*
LOC18776384	XM_020564475.1	XP_020420064.1	*heat stress transcription factor B-2b, transcript variant X2*	*2.28*	*0.035*
LOC18768018	XM_007200293.2	XP_007200355.1	*UDP-galactose transporter 2*	2.28	0.019
LOC18785165	XM_007218813.2	XP_007218875.1	*CDPK-related kinase 4*	*2.25*	*0.033*
LOC18785268	XM_007219503.2	XP_007219565.2	*ethylene-responsive transcription factor ERF106*	*2.25*	*0.043*
LOC18766507	XM_007199633.2	XP_007199695.2	*calcium-transporting ATPase 12, plasma membrane-type*	*2.24*	*0.043*
LOC18769333	XM_007202197.2	XP_007202259.1	*fructose-bisphosphate aldolase, cytoplasmic isozyme*	*2.24*	*0.019*
LOC18778646	XM_020563201.1	XP_020418790.1	*50S ribosomal protein L34, chloroplastic*	*2.20*	*0.044*
LOC18766383	XM_007199596.2	XP_007199658.1	*serine/threonine-protein kinase STY46, transcript variant X1*	*2.20*	*0.023*
LOC18777605	XM_007209252.2	XP_007209314.1	*ethylene-responsive transcription factor 5*	*2.20*	*0.022*
LOC18783057	XM_007217136.2	XP_007217198.1	*non-specific lipid-transfer protein 2*	*2.16*	*0.024*
LOC18785293	XM_007218710.2	XP_007218772.1	*probable aquaporin PIP2-5*	*2.14*	*0.041*
LOC18781811	XM_007215725.2	XP_007215787.2	*biotin carboxyl carrier protein of acetyl-CoA carboxylase*	*2.13*	*0.025*
LOC18776564	XM_007209454.2	XP_007209516.1	*bax inhibitor 1*	*2.12*	*0.047*
LOC18793488	XM_007222228.2	XP_007222290.1	*probable beta-1,4-xylosyltransferase IRX9H*	*2.07*	*0.028*
LOC18786093	XM_007220350.2	XP_007220412.2	*putative lipase ROG1*	*2.06*	*0.036*
LOC18772322	XM_007205075.2	XP_007205137.1	*WRKY transcription factor 44, transcript variant X3*	*2.05*	*0.029*
LOC18788001	XM_007221428.2	XP_007221490.1	*probable WRKY transcription factor 23*	*2.00*	*0.041*

Cont'd

Gene_id	mRNA_accession	Protein accession	Gene descriptions	Log fold change	*p* value
LOC18772307	XM_007205984.2	XP_007206046.1	*zinc finger A20 and AN1 domain-containing stress-associated protein 3*	2.00	0.048
**Down-regulated**					
LOC18777021	XM_007209293.2	XP_007209355.1	*magnesium protoporphyrin IX methyltransferase, chloroplastic*	-4.77	0.049
LOC18789892	XM_007222098.2	XP_007222160.1	*probable pectate lyase 8*	-4.63	0.033
LOC18787444	XM_020557608.1	XP_020413197.1	*glyoxylate/hydroxypyruvate reductase HPR3, transcript variant X3*	-3.29	0.012
LOC18771700	XM_007202680.2	XP_007202742.1	*protein RALF-like 34*	-3.28	0.016
LOC18769359	XM_007202057.2	XP_007202119.1	*calcium uniporter protein 2, mitochondrial*	-3.27	0.002
LOC18790537	XM_007222967.2	XP_007223029.1	*caffeoylshikimate esterase*	-3.23	0.016
LOC18786520	XM_007218309.2	XP_007218371.1	*chalcone--flavonone isomerase*	-3.13	0.020
LOC18781009	XM_007211705.2	XP_007211767.1	*ycf3-interacting protein 1, chloroplastic*	-3.08	0.028
LOC18782204	XM_007215934.2	XP_007215996.1	*30S ribosomal protein 3, chloroplastic*	-3.08	0.026
LOC18768052	XM_020570437.1	XP_020426026.1	*probable ethylene response sensor 1, transcript variant X2*	-3.06	0.009
LOC18786646	XM_007220912.2	XP_007220974.1	*binding partner of ACD11 1*	-3.04	0.005
LOC18768521	XM_007199673.2	XP_007199735.1	*U-box domain-containing protein 15*	-3.01	0.036
LOC18788573	XM_007222706.2	XP_007222768.1	*homogentisate phytyltransferase 1, chloroplastic*	-3.00	0.029
LOC18782078	XM_007214887.2	XP_007214949.1	*receptor protein kinase TMK1*	-2.98	0.035
LOC18786236	XM_020558363.1	XP_020413952.1	*protein IQ-DOMAIN 1, transcript variant X2*	-2.94	0.028
LOC18788709	XM_007226933.2	XP_007226995.1	*subtilisin-like protease SBT1.7*	-2.80	0.014
LOC109948334	XM_020561096.1	XP_020416685.1	*7-deoxyloganetin glucosyltransferase-like*	-2.79	0.043
LOC18766941	XM_007201311.2	XP_007201373.1	*probable 2-oxoglutarate-dependent dioxygenase At5g05600*	-2.79	0.012
LOC18784565	XM_007218152.2	XP_007218214.2	*probable membrane-associated kinase regulator 6*	-2.76	0.041
LOC18782183	XM_007215315.2	XP_007215377.1	*protein farnesyltransferase subunit beta*	-2.73	0.017
LOC109948390	XM_020561186.1	XP_020416775.1	*thioredoxin-like protein CXXS1*	-2.68	0.049
LOC18784663	XM_020556720.1	XP_020412309.1	*probable prolyl 4-hydroxylase 12, transcript variant X1*	-2.64	0.045
LOC18784624	XM_007220161.2	XP_007220223.1	*beta-amylase 7*	-2.61	0.049

Cont'd

**Gene_id**	**mRNA_accession**	**Protein accession**	**Gene descriptions**	**Log fold change**	***p* value**
LOC18788499	XM_007225093.2	XP_007225155.1	*two-component response regulator ARR2, transcript variant X2*	*-2.61*	*0.011*
LOC18783524	XM_007215463.2	XP_007215525.1	*probable fructokinase-6, chloroplastic*	*-2.57*	*0.017*
LOC18767462	XM_007201294.2	XP_007201356.2	*ADP-ribosylation factor-like protein 2*	*-2.57*	*0.045*
LOC18784309	XM_020560648.1	XP_020416237.1	*phytoene synthase 2, chloroplastic*	*-2.49*	*0.024*
LOC18793939	XM_020555471.1	XP_020411060.1	*serine/threonine-protein kinase CTR1, transcript variant X2*	*-2.49*	*0.044*
LOC18767253	XM_007200562.2	XP_007200624.2	*pathogenesis-related protein 1*	*-2.38*	*0.029*
LOC18782207	XM_020558899.1	XP_020414488.1	*WRKY transcription factor 1, transcript variant X1*	*-2.33*	*0.026*
LOC18782906	XM_007215833.2	XP_007215895.1	*membrane-associated progesterone-binding protein 4*	*-2.28*	*0.034*
LOC18782125	XM_020560215.1	XP_020415804.1	*protein phosphatase 2C 16, transcript variant X2*	*-2.26*	*0.038*
LOC18790012	XM_007225054.2	XP_007225116.1	*EIN3-binding F-box protein 1*	*-2.26*	*0.043*
LOC18766258	XM_007201027.2	XP_007201089.1	*homoserine kinase*	*-2.21*	*0.043*
LOC18770881	XM_007203554.2	XP_007203616.1	*4-coumarate--CoA ligase-like 7*	*-2.15*	*0.046*
LOC18786577	XR_002270240.1		*pyruvate kinase 1, cytosolic*	*-2.13*	*0.030*
LOC18783169	XM_007215295.2	XP_007215357.1	*COBRA-like protein 1*	*-2.11*	*0.032*
LOC18767451	XM_007199766.2	XP_007199828.1	*4-hydroxy-3-methylbut-2-enyl diphosphate reductase, chloroplastic*	*-2.05*	*0.033*
